# First records of *Epeus bicuspidatus* and description of a new species of *Epeus* (Araneae, Salticidae) from Vietnam

**DOI:** 10.3897/BDJ.13.e172899

**Published:** 2025-12-01

**Authors:** Duc-Toan Vu, Van-Tang Duong, Dinh-Sac Pham

**Affiliations:** 1 Faculty of Agriculture and Forestry, Tay Bac University, Quyet Tam ward, Son La, Vietnam Faculty of Agriculture and Forestry, Tay Bac University, Quyet Tam ward Son La Vietnam; 2 Vietnam National Museum of Nature (VNMN), Vietnam Academy of Science and Technology (VAST), Hanoi, Vietnam Vietnam National Museum of Nature (VNMN), Vietnam Academy of Science and Technology (VAST) Hanoi Vietnam; 3 Graduate University of Sciences and Technology (USTH), Vietnam Academy of Science and Technology (VAST), Hanoi, Vietnam Graduate University of Sciences and Technology (USTH), Vietnam Academy of Science and Technology (VAST) Hanoi Vietnam

**Keywords:** *
Epeus
*, new species, Salticidae, species diversity, Vietnam

## Abstract

**Background:**

*Epeus* is a genus of jumping spiders (Salticidae) comprising 20 species, four of which have been recorded in Vietnam.

**New information:**

In this paper, we present descriptions and illustrations of two jumping spider species of the genus *Epeus* from Vietnam. *Epeus bicuspidatus* (Song, Gu & Chen, 1988) is recorded from the country for the first time, while *Epeus taybac* Vu & Pham, sp. nov., is described here as new to science. These findings improve the current understanding of *Epeus* diversity in Vietnam, bringing the total number of known species of this genus in the country to six.

## Introduction

The genus *Epeus* Peckham & Peckham, 1885 (Araneae, Salticidae) currently comprises 20 valid species, primarily distributed across Southeast Asia (WSC 2025). Species of this genus are distinguished by several characteristic morphological features, particularly in the male palpal organ, which includes a flattened and elongated cymbium, a characteristic apophysis (CA) directed towards the tibia, a tegular bulb (TB) bearing a tongue-like process and a filiform embolus. The legs are densely covered with setae and bear numerous spines. In females, the epigyne is characterised by long, translucent copulatory ducts (CD) that form several loops (Zabka 1985, Peng and Li 2002, Meng et al. 2015).

To date, four species of *Epeus* have been recorded from Vietnam: *E. glorius*, *E. flavobilineatus*, *E. albus* and *E. phamtri* (Zabka 1985, Patoleta et al. 2020, Tam and Hill 2025, Tam and Thuy 2025, Hill 2025). Despite these records, the genus remains poorly studied in the country and additional species are likely to be discovered through continued fieldwork and taxonomic research.

Between May 2024 and March 2025, field surveys were conducted in Son La, Lao Cai and Quang Tri Provinces of Vietnam. These surveys yielded the first records of *Epeus bicuspidatus* (Song, Gu & Chen, 1988) from Vietnam, along with several specimens exhibiting the general morphological characteristics of the genus *Epeus*, yet differing significantly in key diagnostic traits from all currently described species. These findings represent important new distributional records and contribute to the growing body of knowledge on Salticidae diversity at both national and global scales.

## Materials and methods

All specimens were preserved in 75% ethanol. Female epigyna were cleared in a 10% potassium hydroxide (KOH) solution at room temperature for approximately 12 hours and subsequently stored in small glass vials with their respective specimens. The left male palp was used for morphological descriptions and illustrations. Specimens were examined and illustrated using Olympus BX51 and SZ61 stereomicroscopes, with measurements taken using an Olympus STM4 measuring microscope. Photographs were captured with a Sony A7 IV digital camera mounted on the Olympus BX51 and SZ61 microscopes. Focus-stacked images were produced using Zerene Stacker software. All measurements are reported in millimetres (mm). Leg measurements are presented as total length, with segment lengths given in the following order: femur, patella + tibia, metatarsus and tarsus. Terminology used in the text and figure legends follows Patoleta et al. (2020).

Abbreviations used in the text and figures are as follows: **ALE** anterior lateral eye; **AME** anterior median eye; **CA** cymbial apophysis; **CD** copulatory duct; **CO** copulatory opening; **E** embolus; **EP** epigynal pocket; **FD** fertilisation duct; **GD** glandular duct; **PLE** posterior lateral eye; **PME** posterior median eye; **PTA** proximal tegular apophysis; **TRA** retrolateral tibial apophysis; **S** spermatheca; **SD** sperm duct; **TB** tegular bump; **TL** tongue-like flap.

## Taxon treatments

### Epeus bicuspidatus

(Song, Gu & Chen, 1988)

69EEB478-D5CE-5DDE-9AA9-89DBEBF69AF2

Plexippoides bicuspidatus : Song et al. (1988): 71, figs. 6-8 (description of male).
Epeus bicuspidatus (Song, Gu & Chen, 1988): Peng et al. (1993): 48, figs. 121-124 (transferred from *Plexippoides*); Song et al. (1999): 508, figs. 291 N-O (♂).

#### Materials

- **Type status:** Other material. **Occurrence:** catalogNumber: TBU-ARA-SAL-002.3 to 002.4; individualCount: 2; sex: 1 male, 1 female; lifeStage: adult; occurrenceID: 805D7F94-6204-5C54-A708-9564FA92D6AD; **Taxon:** scientificName: *Epeus bicuspidatus* (Song, Gu & Chen, 1988); order: Araneae; family: Salticidae; genus: Epeus; **Location:** country: Vietnam; countryCode: VN; stateProvince: Son La; locality: Tu Le Commune; verbatimElevation: 1513 m; verbatimLatitude: 21°45′16″N; verbatimLongitude: 104°14′43″E; verbatimCoordinateSystem: WGS84; **Event:** eventDate: 22May 2024; eventRemarks: collected by Duc-Toan Vu
- **Type status:** Other material. **Occurrence:** catalogNumber: VNMN-SAL-079; individualCount: 1; sex: female; lifeStage: adult; occurrenceID: 4E813C9D-1B5A-5E63-8FDF-10FF9DADE4BA; **Taxon:** scientificName: *Epeus bicuspidatus* (Song, Gu & Chen, 1988); order: Araneae; family: Salticidae; genus: Epeus; **Location:** country: Vietnam; countryCode: VN; stateProvince: Quang Tri; locality: Dakrong Nature Reserve; verbatimElevation: 965 m; verbatimLatitude: 16°77′89″N; verbatimLongitude: 106°84′95″E; verbatimCoordinateSystem: WGS84; **Event:** eventDate: 12July 2024; eventRemarks: collected by Dinh-Sac Pham

#### Description

**Male**: Total length 6.91. Carapace 2.55 long, 2.12 wide. Abdomen 3.44 long, 1.48 wide. Eye sizes and interdistances: AME 0.62, ALE 0.29, PME 0.08, PLE 0.26; distance ALE–PME 0.35, ALE–PLE 0.76. Leg measurements: I 6.16 (1.96, 0.91, 1.60, 1.04, 0.65); II 7.34 (2.24, 0.86, 1.51, 1.74, 0.99); III 7.21 (2.05, 0.93, 1.49, 1.78, 0.96); IV 6.93 (1.86, 0.79, 1.64, 1.82, 0.82).

**Female**: Total length 6.94. Carapace 2.78 long, 2.01 wide. Abdomen 3.52 long, 1.36 wide. Eye sizes and interdistances: AME 0.69, ALE 0.38, PME 0.14, PLE 0.31; distance ALE–PME 0.35, ALE–PLE 0.81. Leg measurements: I 7.17 (2.22, 1.03, 1.85, 1.26, 0.81); II 6.70 (2.13, 0.93, 1.73, 1.18, 0.73); III 7.42 (2.46, 0.68, 1.75, 1.71, 0.82); IV 6.75 (2.10, 0.71, 1.63, 1.49, 0.82).

**Figure 1.**
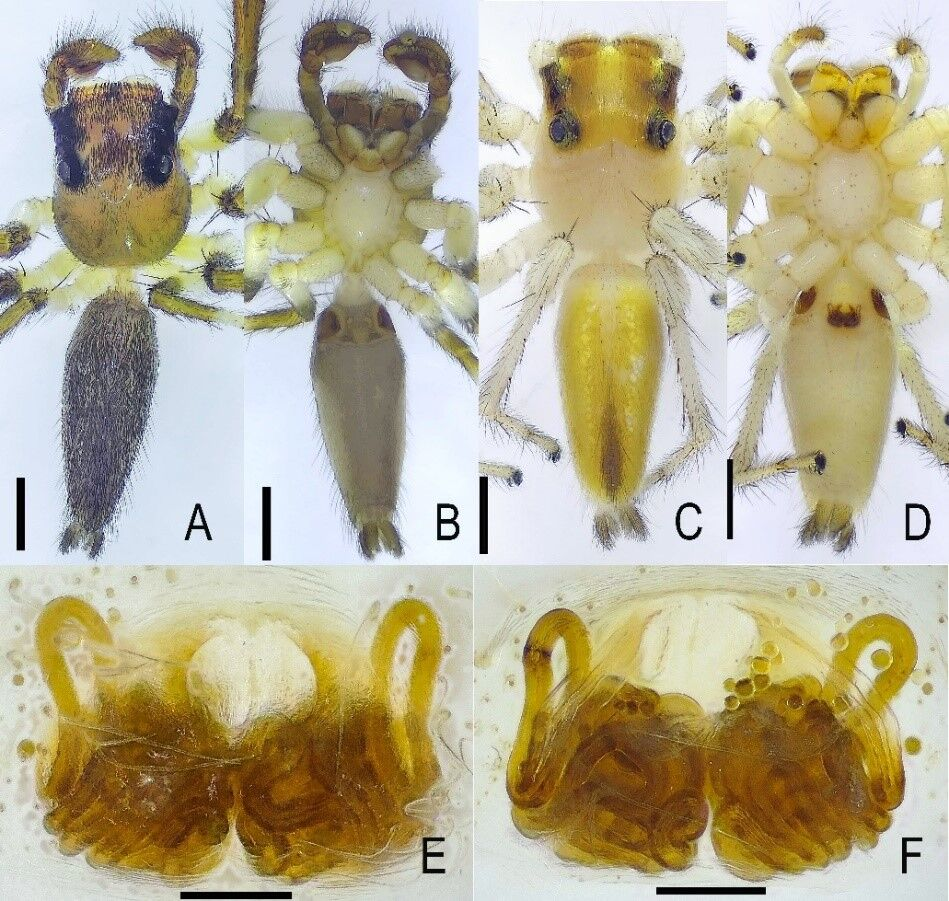
*Epeus bicuspidatus*. **A** Male, dorsal view; **B** Male, ventral view; **C** Female, dorsal view; **D** Female, ventral view; **E** Epigyne, ventral view; **F** Vulva, dorsal view. Scale bars: **A–D** = 1.0 mm; **E–F** = 0.1 mm.

**Figure 2.**
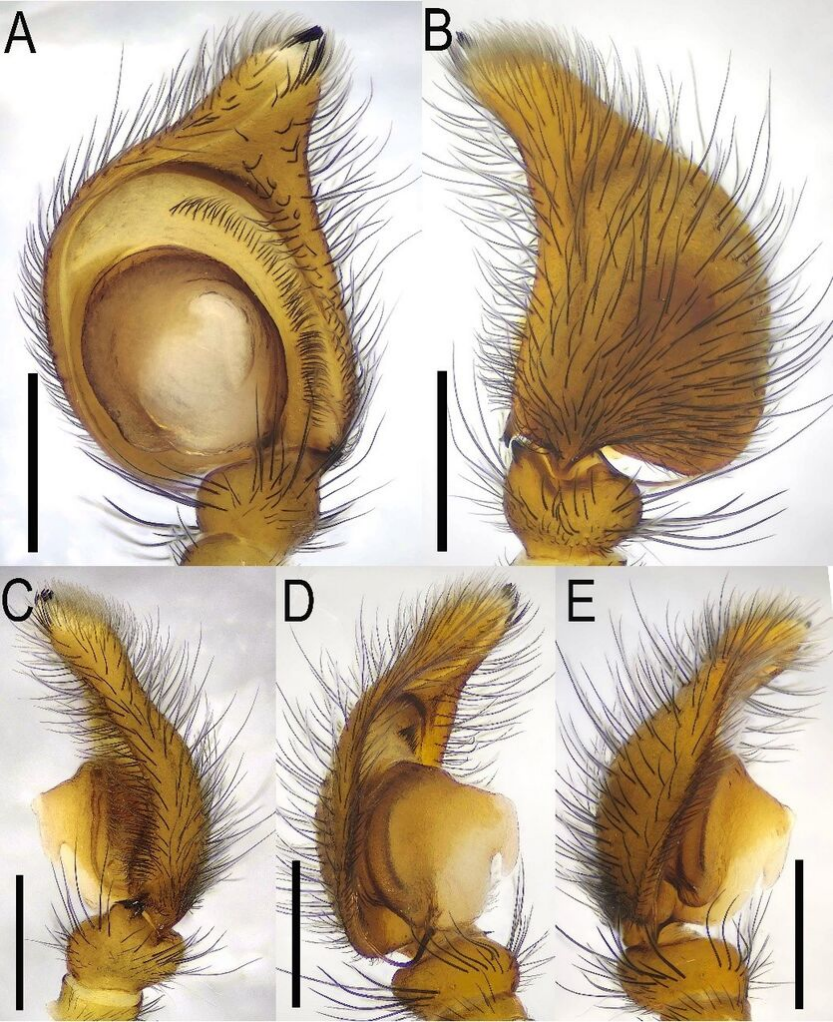
*Epeus bicuspidatus*, left palp habitus. **A** Ventral view; **B** Dorsal view; **C** Retrolateral view; **D–E** Prolateral views. Scale bars: 0.1 mm.

Morphological diagnoses of the male specimens are illustrated in Fig. 1A, B and Fig. 2, while those of the female specimens are depicted in Fig. 1C–F. These closely correspond to the descriptions provided by Peng and Li (2002) and Meng et al. (2015).

### Epeus taybac

Vu & Pham sp. nov.

AE8DB552-37EC-58D0-9CC4-4930DBD07FD5

7BFF4FE7-1CAF-4AED-A627-EE6F46C68A2A

#### Materials

- **Type status:** Holotype. **Occurrence:** catalogNumber: TBU-ARA-SAL-003-1; individualCount: 1; sex: 1 male; lifeStage: adult; occurrenceID: EE56D8CD-D43D-5C2B-AAFB-4E5F20090640; **Taxon:** scientificName: *Epeus taybac* sp. nov.; order: Araneae; family: Salticidae; genus: Epeus; **Location:** country: Vietnam; countryCode: VN; stateProvince: Son La; locality: Muong La Commune, Pieng Village; verbatimElevation: 680 m; verbatimLatitude: 21°34′57″N; verbatimLongitude: 104°04′58″E; verbatimCoordinateSystem: WGS84; **Event:** eventDate: 22May 2024; eventRemarks: collected by Duc-Toan Vu
- **Type status:** Paratype. **Occurrence:** catalogNumber: TBU-ARA-SAL-003-2; individualCount: 1; sex: female; lifeStage: adult; occurrenceID: 068C9472-D849-5CDA-A0AD-6111C93FF431; **Taxon:** scientificName: *Epeus taybac* sp. nov.; order: Araneae; family: Salticidae; genus: Epeus; **Location:** country: Vietnam; countryCode: VN; stateProvince: Son La; locality: Muong La Commune, Pieng Village; verbatimElevation: 680 m; verbatimLatitude: 21°34′57″N; verbatimLongitude: 104°04′58″E; verbatimCoordinateSystem: WGS84; **Event:** eventDate: 22May 2024; eventRemarks: collected by Duc-Toan Vu
- **Type status:** Other material. **Occurrence:** catalogNumber: TBU-ARA-SAL-003-3; individualCount: 1; sex: 1 male; lifeStage: adult; occurrenceID: 6B109DDB-74B5-5ED6-B4F2-CEE7CE57FC12; **Taxon:** scientificName: *Epeus taybac* sp. nov.; order: Araneae; family: Salticidae; genus: Epeus; **Location:** country: Vietnam; countryCode: VN; stateProvince: Son La; locality: Chieng Coi Ward, Na Phum Village; verbatimElevation: 875 m; verbatimLatitude: 21°18′23″N; verbatimLongitude: 103°51′03″; verbatimCoordinateSystem: WGS84; **Event:** eventDate: 11March 2025; eventRemarks: collected by Duc-Toan Vu

#### Description

**Male**. Total length 7.74. Carapace 2.84 long, 2.33 wide. Abdomen 3.62 long, 1.48 wide. Eye sizes and interdistances: AME 0.72, ALE 0.32, PME 0.09, PLE 0.33, ALE-PME 0.34, ALE-PLE 0.81. Legs measurements: I 9.76 (2.81, 1.34, 2.79, 2.01, 0.81), II 7.72 (2.28, 1.12, 2.04, 1.58, 0.70), III 9.04 (2.95, 1.10, 1.91, 2.18, 0.90), IV 7.95 (2.35, 1.00, 1.77, 1.85, 0.98).

**Figure 3.**
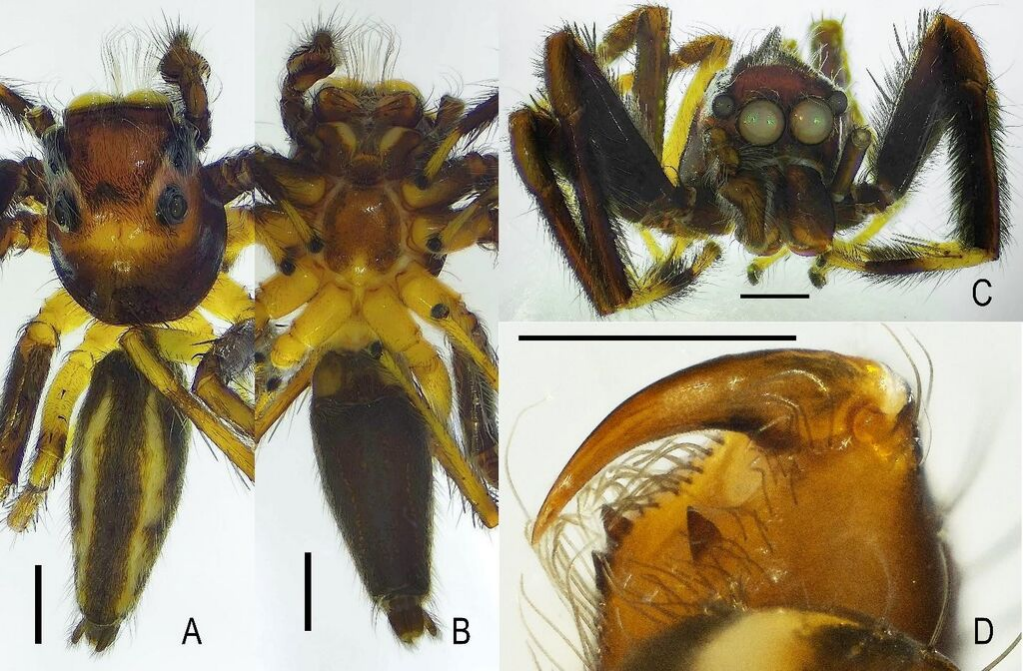
*Epeus taybac* sp. nov., male habitus. **A** Dorsal view; **B** Ventral view; **C** Frontal view; **D** Chelicera, posterior view. Scale bars: 1.0 mm.

The carapace is high and elevated, with a posterior slope, exhibiting a colouration ranging from yellow-brown to dark brown (Fig. 3A). A crest of erect hairs is present along the carapace (Fig. 3C). The sternum is oval, slightly wider posteriorly, brown with a lighter margin. The clypeus is dark brown, covered with brown and white hairs and bristles. Chelicerae, endites and labium are dark brown, chelicerae with two promarginal teeth and one retromarginal tooth (Fig. 3D). Legs are long and slender; legs I, II and III are dark brown and covered with setae, except for the metatarsus and tarsus, which are yellowish-brown, while leg IV is entirely yellowish-brown. The abdomen is slender and cylindrical, with a dark brown dorsum featuring two light-coloured lateral stripes and a dark brown ventral surface.

**Figure 4.**
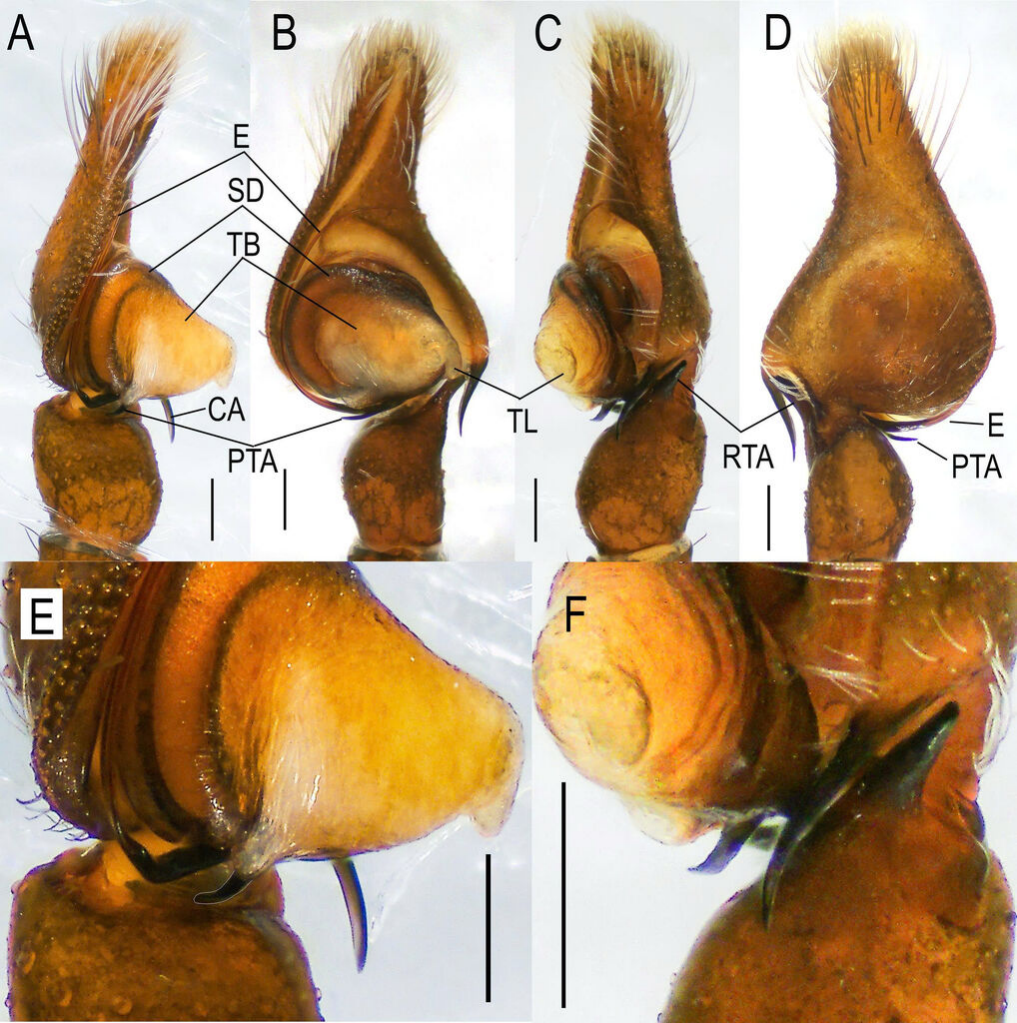
*Epeus taybac* sp. nov., holotype left palp habitus view. **A, E** prolateral view; **B** ventral view; **C, F** retrolateral view; **D** dorsal view. Scale bars: 0.1 mm.

The male palpal organ (Fig. 4) exhibits a filiform embolus that is long and arises at the 6:00 o’clock position, coiling approximately 180°. A well-developed, tongue-like flap is present at a 90° angle on the tegular bump. The proximal tegular apophysis is slender, sharp and curved (Fig. 4A, B and E). In dorsal view, the cymbium is nearly triangular, with the retrolateral edge concave, tapering anteriorly and covered with long white setae. The cymbial apophysis is elongate and slender, with its distal end slightly curved. The retrolateral tibial apophysis is stout, slightly blunt and directed anterodorsally (Fig. 4C and F).

**Female**. Total length 5.50. Carapace 2.62 long, 1.48 wide. Abdomen 2.87 long, 1.32 wide. Eye sizes and interdistances: AME 0.52, ALE 0.16, PME 0.06, PLE 0.21; ALE–PME 0.25, ALE–PLE 0.62. Legs measurements: I 5.58 (1.70, 0.81, 1.57, 1.01, 0.49), II 5.47 (1.69, 0.78, 1.31, 1.08, 0.61), III 6.13 (1.93, 0.68, 1.34, 1.53, 0.65), IV 5.91 (1.78, 0.52, 1.44, 1.46, 0.71).

**Figure 5.**
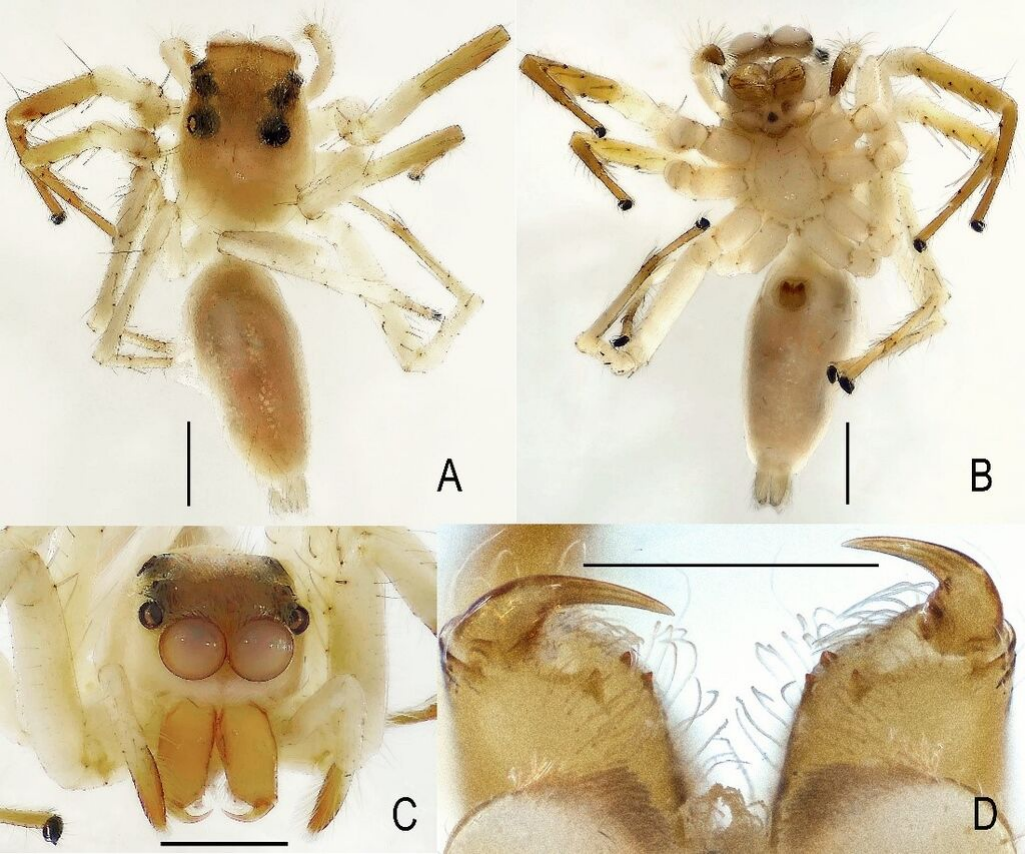
*Epeus taybac* sp. nov., female habitus. **A** Dorsal view; **B** Ventral view; **C** Frontal view; **D** Chelicera, posterior view. Scale bars: 1.0 mm.

**Figure 6.**
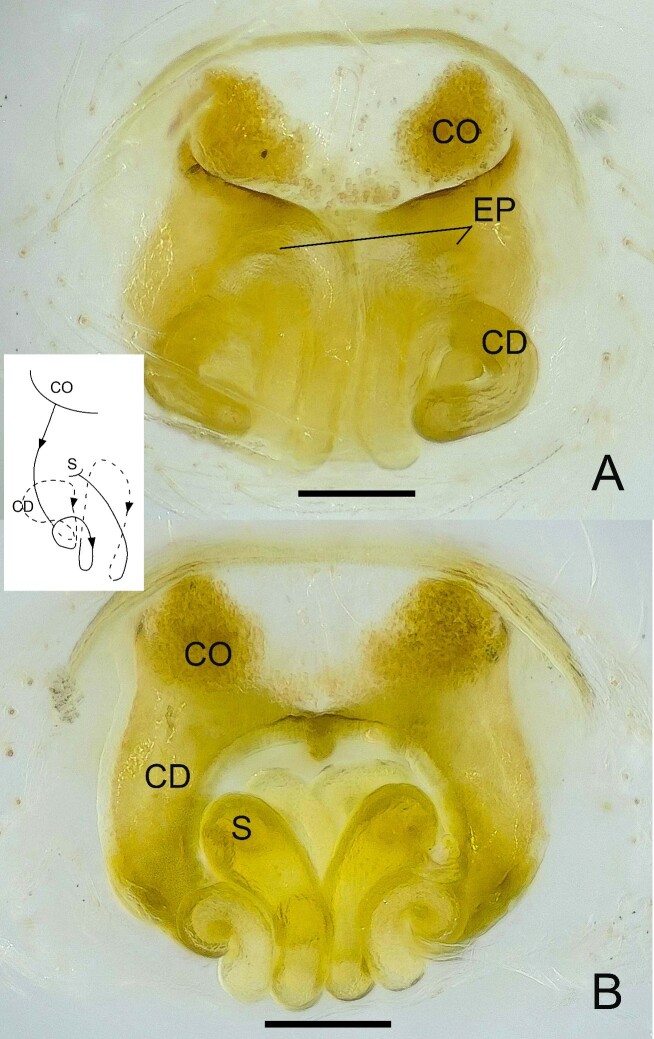
*Epeus taybac* sp. nov. **A** Epigyne, ventral view; **B** Vulva, dorsal view. Scale bars: 0.1 mm.

The carapace is similar in shape to that of the male, exhibiting a pale yellow to yellow-orange colouration. The anterior half of the carapace is covered with colourless and white setae (Fig. 5A). The area surrounding the anterior median eyes is yellow, while the regions around the other eyes are dark brown to black-brown, interspersed with white setae. The clypeus is densely covered with white setae (Fig. 5C). Chelicerae are yellow, whereas the endites, labium and sternum are pale yellow. Legs and palps are predominantly pale yellow, with the exception of the metatarsi and tarsi, which exhibit a yellow-orange hue. The abdomen is slender and dorsally pale yellow, adorned with clusters of white spots arranged along the lateral margins. The ventral surface is pale yellow. The epigynal plate is longer than wide (Fig. 6A), featuring cup-shaped copulatory openings. The internal copulatory ducts are long and expansive, forming numerous loops.

#### Diagnosis

The male genitalia of *Epeus taybac* sp. nov. resemble those of *E. albus*, *E. daiqini* and *E. glorius*. However, *E. taybac* sp. nov. can be distinguished by the following characters: the presence of a proximal tegular apophysis (PTA), which is long, curved and located posterior to the tegular bump (TB) on the patella side (Fig. 4A, B and E); the cymbial apophysis (CA) is long and pointed when viewed ventrally, with the tip slightly curved outwards (Fig. 4B), in contrast to the blunt tip observed in *E. albus* (Tam and Thuy 2025) and considerably longer than the CA of *E. daiqini* (Patoleta et al. 2020), lacking the small serrated apophyses present in *E. glorius* (Zabka 1985). The retrolateral tibial apophysis (RTA) is approximately one-third the length of the tibia, stout, slightly blunt and directed anterodorsally (Fig. 4C and F). Females of the new species are morphologically similar to *E. albus*, sharing wide copulatory openings and a comparable copulatory duct arrangement when viewed ventrally (Fig. 6A). However, *E. taybac* sp. nov. can be differentiated by the more compact arrangement of the copulatory ducts (CD), which form an oval shape in dorsal view (Fig. 6).

#### Etymology

The species is named after its type locality: *taybac* refers to “Tây Bắc,” the Vietnamese name for the north-western region of Vietnam.

## Supplementary Material

XML Treatment for Epeus bicuspidatus

XML Treatment for Epeus taybac

## References

[B13513803] Hill D. E. (2025). Identification of jumping spiders placed in the genus *Epeus* (Araneae: Salticidae: Plexippini: Plexippina). Peckhamia.

[B13510012] Meng Xiang-Wei, Zhang Zhi-Sheng, Shi Ai-Ming (2015). Description of two unknown females of *Epeus* Peckham & Peckham from China (Araneae: Salticidae).. Zootaxa.

[B13510057] Patoleta Barbara Maria, Gardzińska Joanna, Żabka Marek (2020). Salticidae (Arachnida, Araneae) of Thailand: new species and records of *Epeus* Peckham & Peckham, 1886 and *Ptocasius* Simon, 1885. PeerJ.

[B13510048] Peng Xian-Jin, Li Shu-Qiang (2002). A review of the genus *Epeus* Peckham & Peckham (Araneae: Salticidae) from China. Oriental Insects.

[B13513821] Peng X. J., Xie L. P., Xiao X. Q., Yin C. M. (1993). Salticids in China (Arachnida: Araneae).

[B13513851] Song D. X., Gu M. B., Chen Z. F. (1988). Three new species of the family Salticidae from Hainan, China. Bulletin of Hangzhou Normal College.

[B13513843] Song D. X., Zhu M. S., Chen J. (1999). The spiders of China.

[B13513785] Tam T. V., Hill D. E. (2025). A new species of jumping spider of the genus *Epeus* from Vietnam (Araneae: Salticidae: Plexippini). Peckhamia.

[B13513776] Tam T. V., Thuy N. H. K. (2025). First record of *Epeus
albus* Prószyński 1992 in Vietnam (Araneae: Salticidae: Plexippini). Peckhamia.

[B13513860] WSC World Spider Catalog. Version 26. Natural History Museum Bern. http://wsc.nmbe.ch.

[B13513794] Zabka M (1985). Systematic and zoogeographic study on the family Salticidae (Araneae) from Vietnam. Annales Zoologici.

