# Corrigendum to "First records of *Epeus bicuspidatus* and description of a new species of *Epeus* (Araneae, Salticidae) from Vietnam"

**DOI:** 10.3897/BDJ.14.e212593

**Published:** 2026-09-11

**Authors:** Dinh-Sac Pham, Vu Duc Toan

**Affiliations:** 1 Vietnam National Museum of Nature (VNMN), Vietnam Academy of Science and Technology (VAST), Hanoi, Vietnam Vietnam National Museum of Nature (VNMN), Vietnam Academy of Science and Technology (VAST) Hanoi Vietnam; 2 Faculty of Agriculture and Forestry, Tay Bac University, Quyet Tam ward, Son La, Vietnam Faculty of Agriculture and Forestry, Tay Bac University, Quyet Tam ward Son La Vietnam

## Abstract

This corrigendum serves to recognise the official museum depository and formal registration codes for the type specimens of the *Epeus taybac* Vu & Pham, 2025.

## Statement

In a recent publication Vu et al. (2025), the type specimens of *Epeus taybac* Vu & Pham, 2025 (holotype male and paratype female) were listed under internal field/institutional catalogue numbers (TBU-ARA-SAL-003-1 and TBU-ARA-SAL-003-2). However, the explicit designation of the official museum depository and its formal collection/registration codes was inadvertently omitted in the text.

To satisfy the requirements of Article 16.4.2 of the International Code of Zoological Nomenclature (ICZN) and ensure full taxonomic validity on taxonomic platforms such as the World Spider Catalogue (WSC), all type specimens have now been officially deposited in the permanent collection of the Vietnam National Museum of Nature (VNMN), Vietnam Academy of Science and Technology (VAST), Hanoi, Vietnam.

We hereby wish to rectify this by assigning post-publication to update the type specimen repository information for *Epeus taybac* sp. nov. as follows:

In Vu et al. (2025), the depository for the type specimens of *Epeus taybac* sp. nov. was not explicitly specified. The holotype and paratype have been officially deposited in the Vietnam National Museum of Nature (VNMN), Hanoi, Vietnam.

The correct materials details for *Epeus taybac* sp. nov. should read as follows:

**Holotype**: Male (VNMN registration code: VNMN-SAL-079; field code: TBU-ARA-SAL-003-1), VIETNAM: Son La Province, Muong La District, Pieng Village, 21**°**34'57''N, 104**°**04'58''E, elev. 680 m, 22 May 2024, coll. Duc-Toan Vu.

**Paratype**: Female (VNMN registration code: VNMN-SAL-080; field code: TBU-ARA-SAL-003-2), same data as holotype.
